# Diphtheria

**Published:** 1878-12

**Authors:** H. C. Ryals

**Affiliations:** McVille, Ga.


### DIPHTHERIA.

By H. C. Ryals, M.D., McVille, Ga.

Of the peculiar nature and cause of this disease—whether or not it depends upon atmospheric cause or bacterian organisms floating in the air—I have not the means to satisfy myself. That it assumes the magnitude of an epidemic of destructive character, I have, however, abundant evidence. About the first of July last, diphtheria made its appearance in our village, was found to spread as an epidemic, and now prevails to a considerable extent in the counties of Telfair and Montgomery. While to the study of its nature and cause much time may yet be profitably employed, I think any plan of treatment followed by success should certainly be made known. The cause, whatever it may be, is certainly unavoidable, so far as I can observe, in the epidemic of which I write.

From the commencement of the epidemic up to this time, I have treated one hundred and fifteen cases. To the first forty-one were given such remedies as had been advised by eminent physicians contributing to the ATLANTA MEDICAL AND SURGICAL JOURNAL, and other journals of the country. Muriated tincture of iron, chlorate of potassium, quinine, salicylic acid, and carbolic acid were used in these cases, and eleven of the forty-one died, making over twenty-nine per cent. of mortality. Most of these medicines were then laid aside. In fact, none of them were regularly used afterwards in the treatment of the seventy-four cases. Now, while we cannot always be positively certain that recovery following treatment depends upon that treatment, we are, and should be, inclined to prefer that which shows the smallest per cent. of deaths. In these seventy-four cases, all of which recovered, the treatment consisted almost exclusively of sulphur in drachm doses every hour or two, according to urgency of the symptoms. It is not necessary to be very particlar about the quantity. Without weighing, I generally gave to a child three or four years old what will lie on the point of a case-knife, every two hours.

This was the sole treatment, except to have the bowels moved, when necessary, with castor oil and spirit of turpentine, and a flannel around the neck. Nutritious food was taken by all as regularly as could be under the circumstances. The success attending this simple treatment has so impressed the people with its importance that many take sulphur and recover without the aid of a physician at all. I feel perfectly confident of success in the treatment of any ordinary case of diphtheria with this remedy, and believe it will bear the test in any epidemic of the disease that may occur.

These facts, hurriedly given, are contributed to your JOURNAL that others may be induced to test the efficacy of a simple, cheap and readily obtained medicine in the treatment of a dangerous and rapidly fatal disease.

